# Biomimetic Nanosponges Enable the Detoxification of *Vibrio vulnificus* Hemolysin

**DOI:** 10.3390/ijms23126821

**Published:** 2022-06-19

**Authors:** Shuaijun Zou, Qianqian Wang, Peipei Zhang, Bo Wang, Guoyan Liu, Fuhai Zhang, Jie Li, Fan Wang, Beilei Wang, Liming Zhang

**Affiliations:** 1 Department of Marine Biomedicine and Polar Medicine, Naval Special Medical Center, Naval Medical University, Shanghai 200433, China; smmuzsj@163.com (S.Z.); abc_w@163.com (Q.W.); m18817365409@163.com (B.W.); lgy_laurie@aliyun.com (G.L.); zhangfh1105@163.com (F.Z.); 18260199036m@sina.com (J.L.); fanwang1130@126.com (F.W.); 2 Department of Marine Biological Injury and Dermatology, Naval Special Medical Center, Naval Medical University, Shanghai 200052, China; zpp19841113@sina.com

**Keywords:** *Vibrio vulnificus*, nanosponge, VvhA, pore-forming toxins, biodetoxification

## Abstract

*Vibrio vulnificus* (*V. vulnificus*) infection-associated multiple antibiotic resistance has raised serious public health concerns. Recently, nanosponges (NSs) have been expected to provide innovative platforms for addressing antibacterial and drug-resistant challenges by targeting various pore-forming toxins (PFTs). In the present study, we constructed NSs to explore the effects and possible mechanism of recombinant *V. vulnificus* hemolysin (rVvhA)-induced injuries. In vitro, NSs significantly reversed rVvhA-induced apoptosis and necrosis, and improved toxin-induced intracellular reactive oxygen species (ROS) production, adenosine triphosphate (ATP) depletion, and apoptosis signaling pathway disruption. To explore the clinical translation potential of NSs, we established VvhA-induced septicemia and wound infection mouse models, respectively, and further found NSs could notably attenuate rVvhA-induced acute toxicity and septicemia-associated inflammation, as well as local tissue damage. In a conclusion, NSs showed excellent protective effects against rVvhA-induced toxicity, thus providing useful insights into addressing the rising threats of severe *V. vulnificus* infections.

## 1. Introduction

*Vibrio vulnificus* (*V. vulnificus*) is an alkalophilic, halophilic, rod-shaped, gram-negative bacterium of the *Vibrio* genus [1,2]. Exposure to *V. vulnificus*-contaminated seawater usually results in hemorrhagic necrotic skin lesions and secondary sepsis, while ingestion of contaminated raw or undercooked seafood mainly leads to primary septicemia [3,4,5]. Septicemia caused by *V. vulnificus* exerts high mortality (>50%), especially in patients with chronic liver diseases or immunocompromising conditions [6,7,8,9]. Currently, combination antibiotic therapy is the first choice for *V. vulnificus* infections and includes doxycycline, third-generation cephalosporin, fluoroquinolone, and trimethoprim-sulfamethoxazole, as well as aminoglycosides [10]; however, due to the excessive use of antibiotics in humans, agricultural and aquaculture systems, multiple antibiotic resistance has emerged in up to 50% of *V. vulnificus* strains in various countries, which raises serious public health and economic concerns [10,11]. Thus, the development of alternative agents is urgently needed.

*V. vulnificus* hemolysin (VvhA) is a pore-forming toxin (PFT) encoded by *vvhA*, which is a key virulence factor in the pathogenesis of *V. vulnificus* infections. Earlier studies [12,13] have shown that local and systemic administration of VvhA in animal models reproduces the same clinical and pathological manifestations as live *V. vulnificus*, and purified VvhA can induce lethal effects in vivo, including hypotension, tachycardia, and pulmonary damage; moreover, VvhA may also contribute to pathogen invasion, vasodilatation and septic shock [14,15,16,17]. These results indicate that VvhA should be a logical target for the development of alternative therapeutics to combat *V. vulnificus* infection. More importantly, targeting the VvhA strategy could effectively prevent drug resistance since it exerts less selective pressure on pathogens themselves; however, the cellular targeting sites of VvhA remain controversial and unclear [16]. For example, the cholesterol on cell membranes was believed to be the receptor for VvhA, while some other reports suggested that VvhA localized on both cholesterol and glycan moieties rich membrane domain and other membrane domains [18,19]; conversely, the molecular mechanism of cell damage after the interaction between VvhA and the host cell during *V. vulnificus* infection has also not been clarified yet. For example, some studies demonstrate that VvhA induced NF-κB-dependent mitochondrial cell death via lipid raft-mediated reactive oxygen species (ROS) production [20]; some other studies proposed that VvhA induced autophagy upregulation through the lipid raft-mediated c-Src/NOX signaling pathway and ERK/eIF2α activation [18]. Collectively, structure-specific PFT-targeting strategies or some signal pathway inhibitors would be insufficient to thoroughly combat VvhA-induced injuries.

Recently, Hu et al. [21,22] proposed a broad-spectrum detoxification strategy, known as “nanosponges (NSs)”, which focused on the whole membranes of affected host cells instead of targeting specific structures. Wrapped with red blood cell (RBC) membranes, NSs provide sufficient, intact, and natural targets for PFTs to bind with, and divert them away from the host cells; moreover, the natural membrane shells endow NSs with lower immunogenicity and prolonged circulation time in vivo [23]. Currently, the NS platform has been proven to be effective against injuries from gram-positive pathogen-derived toxins [24,25]; however, fewer studies have been conducted to investigate the protective effects and possible molecular mechanisms of NSs against gram-negative pathogen-derived PFTs.

In this study, we tried to construct NSs by fusing RBC vesicles onto poly (DL-lactide-co-glycolide) (PLGA) nanoparticles (NPs), which are the biocompatible polymers approved by the US Food and Drug Administration [26,27,28]. In vitro, we chose human pulmonary artery endothelial cells (HPAECs) as effector cells, which are the most common target cells in *V. vulnificus*-related sepsis. Then, we examined the effects and possible mechanisms of NSs on toxin-induced intracellular oxidative stress, adenosine triphosphate (ATP) depletion, and the activation of apoptosis signaling pathways. In vivo, we respectively constructed VvhA-induced septicemia and wound infection mouse models to simulate clinical cases, then tested the protective effects of NSs against VvhA by recording the animal survival rates, inflammatory cascades as well as lung and tissue damage.

## 2. Results

### 2.1. Characterization of NSs

**Figure 1:**
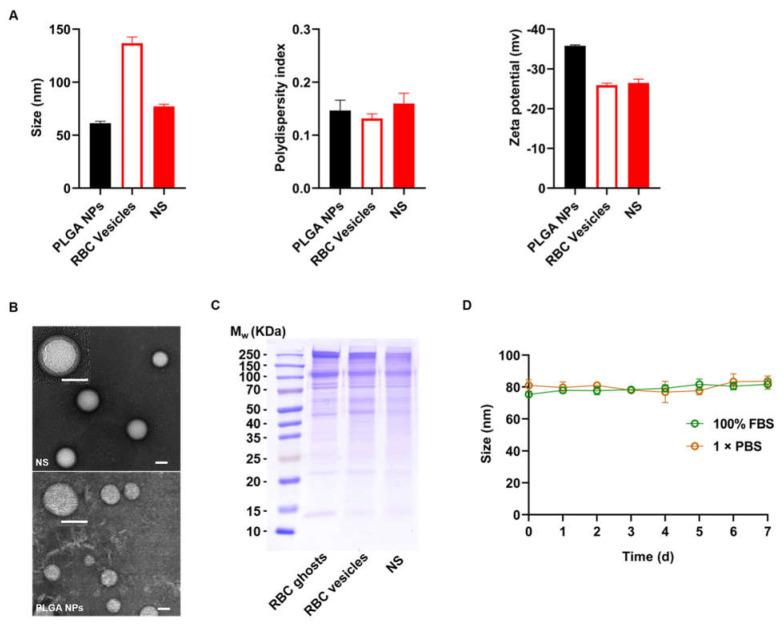
Characterization of NSs. (**A**) Hydrodynamic size, polydispersity index and zeta potential of PLGA NPs, RBC vesicles, and NSs. (**B**) TEM image showing the structure of PLGA NPs (lower) and NSs (upper). (Scale bar: 50 nm.) (**C**) Proteins in RBC ghosts, RBC vesicles, and NSs were examined by SDS-PAGE. (**D**) NSs were suspended in 1 × PBS or 100% FBS, and the hydrodynamic size was monitored for 7 d.

To characterize the hydrodynamic size and zeta potential, PLGA NPs and RBC-derived NSs were measured by dynamic light scattering (DLS). The results revealed that the diameters of NPs increased from 61.3 ± 1.1 to 77.2 ± 1.4 nm with a narrow size distribution, corresponding to the bilayered membrane coating on a polymeric core; moreover, the zeta-potential of the PLGA NPs decreased from −35.8 ± 0.3 to −26.5 ± 0.6 mV, and the latter one corresponds to the potential of RBC vesicles (Figure 1A). These results demonstrated fusion between the NP cores and the RBC membranes. To characterize the nanostructures, the resulting NS sample was dried and examined by transmission electron microscopy (TEM). Compared to bare NPs, the resulting NSs exhibited a typical core-shell structure with PLGA cores enwrapped with thin membrane shells, verifying the formation of NSs (Figure 1B). To further characterize the protein contents retained on NSs, the resulting NS sample was examined by SDS-PAGE. The results showed that most membrane proteins were retained on NS shells, which were consistent with those of RBC ghosts and extruded RBC vesicles with a negligible loss during preparation (Figure 1C). Finally, NSs were suspended in 1 × phosphate-buffered saline (PBS) and 100% fetal bovine serum (FBS) and showed only negligible increases in particle size over 7 d at 4 °C, confirming the excellent stability of the prepared NSs (Figure 1D).

### 2.2. Inhibitory Effect of NSs on Cell Viability and Apoptosis Induced by Recombinant VvhA (rVvhA)

**Figure 2:**
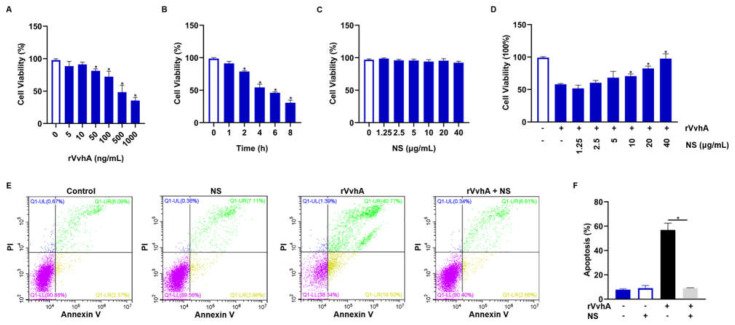
Inhibitory effects of NSs on rVvhA-induced cell death and apoptosis in HPAECs. (**A**) Dose-response of cell viability in HPAECs treated with rVvhA for 4 h. (**B**) Time response of HPAECs treated with rVvhA (500 ng/mL) over a period of 8 h (0, 1, 2, 4, 6, 8 h). (**C**) HPAECs were treated with increasing concentrations of NSs (0, 1.25, 2.5, 5, 10, 20 and 40 μg/mL) for 4 h. (**D**) HPAECs were treated with increasing concentrations of NSs (0, 1.25, 2.5, 5, 10, 20 and 40 μg/mL) in the presence of rVvhA (500 ng/mL) for 4 h. (**E**,**F**) HPAECs were treated with PBS, NSs (40 μg/mL), rVvhA (500 ng/mL), rVvhA (500 ng/mL) plus NSs (40 μg/mL) for 4 h, respectively. The percentages of total apoptotic cells were measured and quantified by using Annexin V/PI staining and flow cytometry. (The data represent the means ± SE. *n* = 3. * *p* < 0.05).

After expression and purification of rVvhA (Figures S1 and S2) [29], we then used HPAECs as target cells to investigate the protective effect of NSs against VvhA-induced cytotoxicity. According to the dose-effect and time-effect relationship of the toxin (Figure 2A,B), we chose the optimal dose of rVvhA (500 ng/mL, the dose producing 50% inhibition) and exposure time (4 h) for subsequent detoxification study. rVvhA-induced cytotoxicity was significantly inhibited by NSs (10–40 μg/mL), and 40 μg/mL NSs almost completely reversed the cytotoxicity of rVvhA, while NSs exerted no effect on cell viability (Figure 2C,D). Similar detoxification effects of NSs (40 μg/mL) were also observed in the context of rVvhA (500 ng/mL)-induced cell apoptosis (Figure 2E,F).

### 2.3. NSs Inhibited ROS Production and ATP Depletion Induced by rVvhA

**Figure 3:**
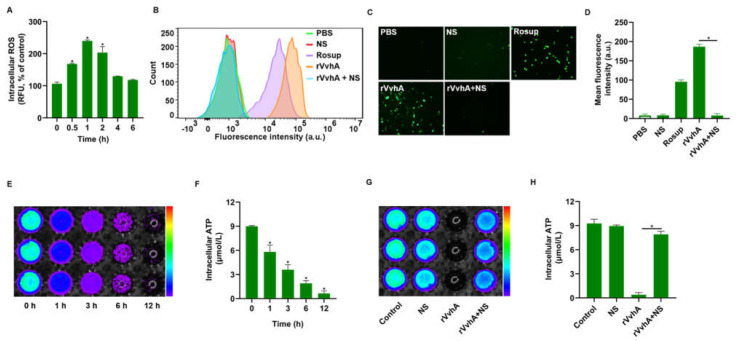
Inhibitory effects of NSs on rVvhA-induced ROS production and ATP reduction in HPAECs. (**A**) Time response of ROS production in HPAECs treated with rVvhA (500 ng/mL). (**B**) Flow cytometry results showing ROS production in HPAECs with various treatments. (**C**) ROS production (green) was visualized by fluorescence microscopy in HPAECs treated with PBS, NSs (40 μg/mL), rVvhA (500 ng/mL), rVvhA (500 ng/mL) plus NSs (40 μg/mL), respectively. (Original magnification × 200). (**D**) The quantification of ROS production in (**C**). (**E**,**F**) Time response of ATP levels in rVvhA-stimulated cells over a period of 12 h (0, 1, 3, 6, 12 h) were examined by a luminometer and bioluminescence imaging. (**G**,**H**) Analysis of ATP levels for 12 h in HPAECs treated with PBS, NSs (40 μg/mL), rVvhA (500 ng/mL), rVvhA (500 ng/mL) plus NSs (40 μg/mL), respectively, as detected by a luminometer and bioluminescence imaging. (The data represent the means ± SE. *n* = 3. * *p* < 0.05).

It was reported that oxidative stress and intracellular ATP depletion were associated with cell necrosis and apoptosis [30,31]; thus, we measured intracellular ROS and ATP levels in this study. As shown in Figure 3A, rVvhA (500 ng/mL) induced a significant increase in intracellular ROS levels, which reached a peak at 1 h; however, NS treatment (40 μg/mL) completely attenuated rVvhA-induced ROS production. Concretely, fluorescence detection via flow cytometry revealed decreased fluorescence in NS-treated cells, and ROS-related fluorescence was quantitively weak under a fluorescence microscope after treatment (Figure 3B–D), indicating that NSs could prevent toxin-induced oxidative stress. Since ATP depletion is a terminal outcome of oxidative stress injuries in mitochondria, we further explored the effects of NSs on intracellular ATP depletion. Our results showed that rVvhA (500 ng/mL) caused a reduction in intracellular ATP in a time-dependent manner, and ATP was reduced by over 90% over a period of 12 h (Figure 3E,F); however, NSs also substantially prevented toxin-induced ATP depletion after 12 h (Figure 3G,H). Accordingly, we demonstrated that NSs may exert protective effects against rVvhA by improving oxidative stress and mitochondrial injury.

### 2.4. NSs Inhibit PKC/ERK/JNK and NF-κB Pathway Activation Induced by rVvhA

**Figure 4:**
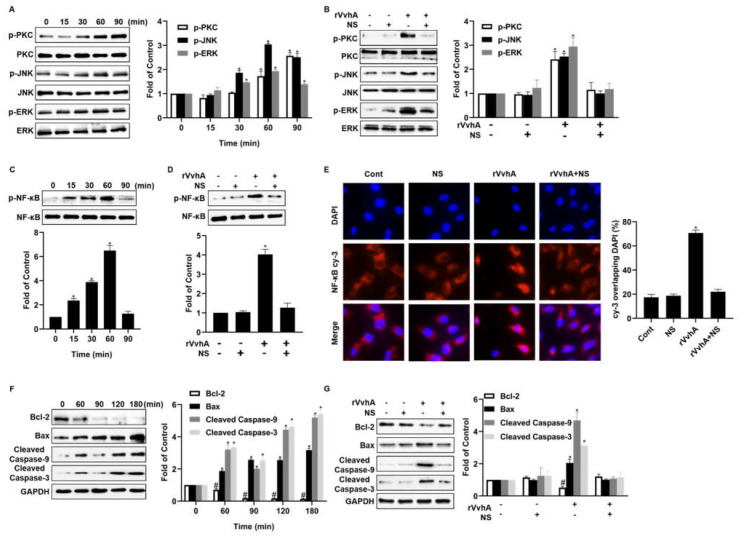
NSs inhibited rVvhA-induced activation of the PKC/JNK/ERK and NF-κB pathways. (**A**) The phosphorylation of PKC/JNK/ERK in HPAECs treated with rVvhA (500 ng/mL) was examined by Western blotting. The data represent the means ± SE. *n* = 3. * *p* < 0.05 versus 0 min. (**B**) The phosphorylation of PKC/JNK/ERK in HPAECs treated with NSs (40 μg/mL) and rVvhA (500 ng/mL) for 60 min. The data represent the means ± SE. *n* = 3. * *p* < 0.05 versus Cont. (**C**) The phosphorylation of NF-κB in HPAECs treated with rVvhA (500 ng/mL) was examined by Western blotting. The data represent the means ± SE. *n* = 3. * *p* < 0.05 versus 0 min. (**D**) The phosphorylation of NF-κB in HPAECs treated with NSs (40 μg/mL) and rVvhA (500 ng/mL) for 60 min. The data represent the mean ± SE. *n* = 3. * *p* < 0.05 versus Cont. (**E**) Nuclear accumulation of NF-κB was examined after 60 min by immunostaining with a Cy3-labeled NF-κB p65 antibody (Original magnification × 400). (**F**) HPAECs were incubated with rVvhA (500 ng/mL) for 180 min, and then Bcl-2 and Bax were analyzed by Western blotting. The data represent the means ± SE. *n* = 3. * *p* < 0.05 versus 0 min, # *p* < 0.05 versus 0 min. (**G**) The expression of Bcl-2 and Bax was measured in the presence of NSs. The data represent the means ± SE. *n* = 3. * *p* < 0.05 versus Cont, # *p* < 0.05 versus Cont.

According to previous studies, VvhA exposure can activate PKC/JNK/ERK phosphorylation, stimulate lipid raft-mediated ROS production, and ultimately cause NF-κB-dependent cell apoptosis in intestinal epithelial cells [20,32]. We, thus, measured the protein expression of these signaling pathways in HPAECs to explore the intracellular changes and protective effects of NSs against VvhA. In our study, we confirmed that rVvhA could stimulate PKC phosphorylation and induce the phosphorylation of both JNK and ERK from 30 to 90 min (Figure 4A), and these events were responsible for the activation of the transcription factor NF-κB; however, NSs significantly blocked rVvhA-induced PKC/JNK/ERK phosphorylation (Figure 4B). Next, we examined the effect of rVvhA on the activation of the NF-κB pathway, which is a direct transcriptional target of the apoptotic signaling pathway. Our results showed that rVvhA (500 ng/mL) increased the phosphorylation level of NF-κB from 15 min to 60 min (Figure 4C); however, NSs notably attenuated toxin-induced NF-κB signaling pathway activation (Figure 4D). A similar inhibitory effect of NSs on NF-κB accumulation in the nucleus was also confirmed by immunofluorescence staining (Figure 4E). Finally, the expression of the main apoptosis factors, including Bcl-2, Bax, Caspase-9, and Caspase-3, was measured, and these factors play key roles in mitochondrial pathways. Our results revealed that rVvhA altered the Bcl-2/Bax ratio from 60 to 180 min, and these proteins are two NF-κB-dependent apoptosis-determining factors (Figure 4F). Consistently, rVvhA also stimulated caspase-9/-3 activation while promoting apoptotic cell death; however, NSs notably reversed VvhA-mediated changes in mitochondrial apoptotic indicators (Figure 4G).

### 2.5. In Vivo Neutralization of rVvhA by NSs

**Figure 5:**
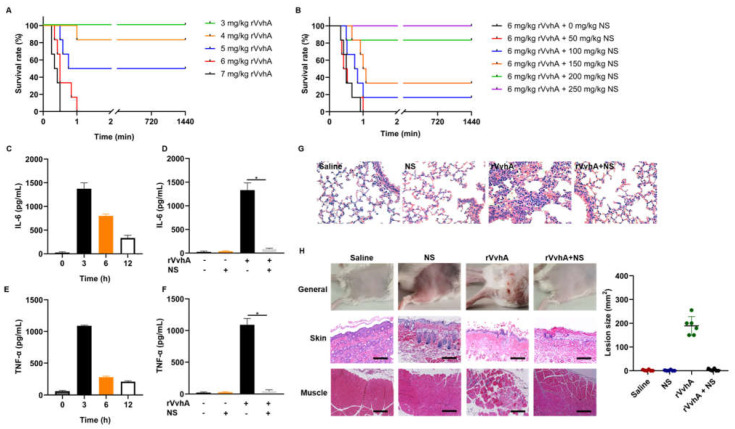
Protective effects of NSs in vivo. (**A**) Different doses of rVvhA (3, 4, 5, 6, 7 mg/kg) were injected intravenously, and mouse mortality was recorded. (**B**) A lethal dose of rVvhA (6 mg/kg) was injected via the tail vein, and different doses of NSs (0, 50, 100, 150, 200, 250 mg/kg) were immediately administered for treatment. (**C**,**E**) Mice were exposed to sublethal doses of rVvhA (3.7 mg/kg), and inflammatory factors, including IL-6 and TNF-α, were examined at predetermined times (0, 3, 6, 12 h). (**D**,**F**) NSs (200 mg/kg) were used to neutralize rVvhA (3.7 mg/kg) for 3 h in vivo, and then IL-6 and TNF-α in the blood were measured. (**G**) rVvhA (3.7 mg/kg) was used to stimulate mice for 3 h with or without NSs (200 mg/kg), and the lungs were harvested for hematoxylin and eosin (H&E) staining and pathological observations. Untreated mice and mice injected with NSs were used as controls. (Original magnification × 400) (**H**) rVvhA (30 μg) was injected subcutaneously to imitate local infections, and NSs (50 mg/kg) were used for treatment. After 3 d, the injection sites were measured and photographed, and then the topical skin and muscle tissues were harvested for H&E staining and pathological observations. (Scale bar: 50 μm. The data represent the means ± SE. *n* = 6. * *p* < 0.05).

To examine the systemic detoxification effects of NSs against rVvhA in vivo, we first analyzed rVvhA-induced acute toxicity by injecting various amounts of rVvhA via the tail vein. Our results showed that the mortality of mice increased with increasing doses of rVvhA (Figure 5A). The survival curve shown in Figure 5B revealed that NS treatment dose-dependently improved the survival of mice that received lethal doses of rVvhA. When the dose of NSs exceeded 200 mg/kg, the survival rate was greater than 80%. Then, NSs (200 mg/kg) were used to examine the reduction in inflammatory cascade reactions caused by rVvhA (3.7 mg/kg, 0.75 × LD_50_, a rational dose to trigger inflammatory and lung damage without lethality). NSs dramatically reversed rVvhA-induced interleukin-6 (IL-6) and tumor necrosis factor-α (TNF-α) accumulation after 3 h (Figure 5C–F). NSs also apparently improved rVvhA-induced lung damage, such as typical hemorrhagic injuries, neutrophil aggregation, and alveolar structural changes (Figure 5G).

To examine the local detoxification effects of NSs against rVvhA in vivo, we further examined the effects of NSs (50 mg/kg) on a rVvhA-induced wound infection mouse model. Histological analyses showed that rVvhA induced obvious reddish ecchymosis and ulcerations on topical skin at 3 d, with typical edema, cell death and inflammation in the skin and muscles, while NS treatment significantly alleviated this tissue damage, confirming the local detoxification effects of NSs (Figure 5H).

## 3. Discussion

*V. vulnificus* is a highly fatal human pathogen that is increasingly being considered an emerging pathogen associated with public health concerns [33]; however, current antibiotic resistance combined with a lack of broad-spectrum virulence factor-targeting strategies highlights the need for innovative research into alternative strategies to combat diseases [10]. In this study, a stable and biocompatible biomimetic nanodetoxification system was developed to neutralize VvhA for the first time. Specifically, NSs showed significant inhibitory effects on not only VvhA-induced apoptosis/necrosis but also toxin-induced septicemia and wound infections. In contrast to traditional strategies, NSs prevent the recognition and interaction between toxins and cells by absorbing and neutralizing VvhA toxins regardless of their specific structures, and further avoiding customization of inhibitors against VvhA-activating cell signaling molecules in treatment. Our work may offer significant potential for the treatment of severe *V. vulnificus* infections.

Multiple-organ failure caused by *V. vulnificus*-associated sepsis always begins with pulmonary dysfunction, which is characterized by increased vascular permeability due to pulmonary endothelial cell damage [34,35,36,37]. We, thus, used HPAECs as target cells to investigate the protective effect of NSs against VvhA-induced cytotoxicity. We first found that NSs could dramatically improve VvhA-induced apoptosis and necrosis in HPAECs. Since oxidative stress and intracellular ATP depletion are associated with necrosis and apoptosis [38,39,40], we further explored the effects of NSs on intracellular ROS production and ATP levels. Our results confirmed that NSs could prevent toxin-induced oxidative stress; moreover, we also found that NSs could apparently improve toxin-induced ATP depletion, which is a terminal outcome of oxidative stress injuries in mitochondria [38,40]. Accordingly, the above results revealed that NSs may exert protective effects against VvhA-induced apoptosis and necrosis by improving oxidative stress and mitochondrial injury. Then, we further explored the effects of NSs on various cell signaling pathways related to oxidative stress and mitochondrial injury. We confirmed that VvhA could induce NF-κB-dependent cell apoptosis via ROS production through the distinct activation of PKC/JNK/ERK in HPAECs. Additionally, NS treatment significantly blocked the activation of the above signaling pathways, as well as apoptosis-related proteins, confirming the protective mechanism of NSs on molecular levels. In previous studies [21,24,41,42,43], NSs were indicated to bind and neutralize toxins via lipid membrane interactions with a high natural affinity and prevent host-toxin interactions. Thus, we hypothesize that the protective mechanism of NSs against VvhA-induced cell apoptosis and necrosis may be associated with toxin sequestration and blocking toxin-host cell interactions, thus radically avoiding the downstream activation of toxin-induced cell signaling disruptions, as well as further cellular injuries. Therefore, for VvhA-induced sepsis, especially pulmonary dysfunction, the sequestration of VvhA by NSs can exert broadly protective effects without consideration of customization of inhibitors against disease-related cell signaling molecules in clinical trials.

To explore the clinical translation potential of NSs, we further established VvhA-induced septicemia mouse models and tested the systemic detoxification effects of NSs against VvhA in vivo. Our work showed that isolated VvhA toxins were fatal but NS injection significantly reversed VvhA-induced mortality, inflammation, and target organ damage, demonstrating that VvhA was indeed a vital virulence factor and NSs exerted significant anti-inflammatory and detoxification effects in vivo. In addition, given that *V. vulnificus* infection often occurs in exposed skin wounds [44], and VvhA can reproduce the same clinical and pathological manifestations as live *V. vulnificus*, we also subcutaneously injected VvhA into mice to simulate local wound infections. Similarly, we found that NSs could alleviate VvhA-induced skin and muscle lesions. These results indicated that NSs have great potential for treating local or systematic *V. vulnificus* infections clinically; however, many challenges should be tackled in preclinical trials [45,46]. First, immune responses are still very critical, and blood-type dependent biological applications may be a good strategy since NSs exert effects via natural RBC membranes. Second, the long-term compatibility of NSs should be further confirmed in large animal models (e.g., primates) before human trials [47]. Finally, the challenge of batch-to-batch quality control should also be addressed before scale-production. Overall, there is still a long way to go before practical application.

## 4. Materials and Methods

### 4.1. Cells, Animals and Reagents

HPAECs were obtained from Xinyu Biotechnology (Shanghai, China) and cultured at 37 °C in a humidified atmosphere of 5% CO_2_ in Roswell Park Memorial Institute 1640 (RPMI 1640) medium containing 10% FBS and antibiotics. All the animal experiments were performed according to the Guide for the Care and Use of Laboratory Animals and were approved by the Medical Ethics Committee of Naval Medical University. FBS, PBS, tris buffered saline tween (TBST) and 0.25% (*w*/*v*) trypsin−0.03% (*w*/*v*) ethylenediaminetetraacetic acid (EDTA) solution were purchased from Grand Island Biological Co. (New York, NY, USA). RPMI 1640 medium was purchased from Solarbio Co. (Beijing, China). Cell-counting (CCK-8) kit was purchased from Dojindo Molecular Technologies, Inc. (Rockville, MD, USA). ROS and ATP detection kits were purchased from Beyotime Biotechnology (Shanghai, China). Carboxy-terminated 50:50 PLGA (Mw 48 000, 0.67 dL/g) was purchased from Lactel Co. (Birmingham, UK). Monoclonal antibodies against PKC (#2056), phosphorylated PKC (p-PKC, #9379), NF-κB (#8242), p-NF-κB(#3033), Bcl-2 (#), caspase-3 (#9664) and caspase-9 (#9509) were purchased from Cell Signaling Technology (Shanghai, China). Monoclonal antibodies against JNK (#AF1048), phosphorylated JNK (p-JNK, #AF1762), ERK (#AF1051), phosphorylated ERK (p-ERK, #AF1891) and Bax (#AF1270) were purchased from Beyotime Biotechnology (Shanghai, China). Tryptone and yeast extract were purchased from Thermo Fisher Scientific (Oxoid, Waltham, MA, USA). All other reagents were of analytical grade, commercially available, and used as received.

### 4.2. Preparation and Characterization of NSs

#### 4.2.1. Preparation of RBC Ghosts and Vesicles

RBC ghosts were prepared as described previously with slight modifications [48]. First, whole blood was collected from male ICR mice (25–30 g) via a cardiac puncture protocol [49]. Then, the cells were washed three times with cold 1 × PBS (pH 7.4) to remove plasma and the buffy coat. Sequentially, to obtain RBC ghosts, the sediments were repeatedly resuspended in 0.25 × PBS for 30 min followed by centrifugation at 14,000 rpm for 20 min at 4 °C until a white pellet was obtained. Finally, to obtain RBC-derived vesicles, the collected RBC ghosts were sonicated for 3 min at a frequency of 45 kHz and power of 200 W (SB-5200DT bath sonicator, Scientz, Ningbo, China) and then serially extruded through 400 nm and 200 nm polycarbonate porous membranes (Avanti mini extruder, Avanti Polar Lipids, Birmingham, UK).

#### 4.2.2. Preparation of PLGA Cores and NSs

PLGA NPs were prepared via the nanoprecipitation method [50]. Briefly, the PLGA polymer was first dissolved in 1 mL of acetone solution (5 mg/mL) and then injected into 3 mL of distilled water. The mixture was subsequently stirred for 1 h and then placed into a vacuum for complete evaporation of the acetone. The resulting NPs were examined by DLS. RBC vesicles and PLGA NPs were mixed at a 1:1 ratio (*w*/*w*) and then sonicated in a bath sonicator for 2 min. As a result, NSs were formed by sequentially extruding the mixture through 400 nm, 200 nm, and 100 nm polycarbonate porous membranes 12 times.

#### 4.2.3. Characterization of NSs

First, the hydrodynamic size and zeta potential of RBC-derived NSs were measured by DLS. Then, the NS structure was examined by TEM with 1% uranyl acetate for negative staining. Additionally, the membrane protein characteristics of RBC ghosts, RBC vesicles, and NSs (with an equivalent protein concentration of 1 mg/mL) were examined by SDS-PAGE on a 6–12% Bis-Tris gel that was run at 150 V for 1 h. The resulting gels were stained with Coomassie Blue (Sangon, Shanghai, China) for visualization. Finally, the stability of NSs was measured by DLS in both PBS and 100% FBS at 4 °C for 7 d.

### 4.3. Cell Viability and Apoptosis/Necrosis Detection in HPAECs

HPAECs were grown on 96-well plates and synchronized in the G_0_/G_1_ phase by culture in a serum-free medium for 24 h. Then, various doses of rVvhA (0, 5, 10, 50, 100, 500, 1000 ng/mL) were added and incubated for an additional 4 h, after which cell viability was analyzed using a CCK-8 kit. Subsequently, the time-dose effect of the toxin was also examined using rVvhA (500 ng/mL, the dose producing 50% inhibition) to treat HPAECs. For toxin neutralization, various doses of NSs (0, 1.25, 2.5, 5, 10, 20, 40 μg/mL) were added and incubated with rVvhA (500 ng/mL) for 30 min and then centrifuged at 10,000 rpm for 10 min. The resulting supernatant was harvested and used to treat cells for 4 h, after which cell viability was examined. A similar detoxification effect of NSs was also examined by measuring cell apoptosis with flow cytometry (FACSCalibur, BD, New York, NY, USA). First, NSs (40 μg/mL) were incubated with rVvhA (500 ng/mL) for 30 min and then centrifuged at 10,000 rpm for 10 min. Subsequently, cells were treated with the supernatants for 4 h and processed with an Annexin V and PI staining kit (Beyotime, Shanghai, China). PBS and NSs were used as controls.

### 4.4. Intracellular ROS and ATP Detection

To quantify intracellular ROS levels, HPAECs were grown on 6-well plates and treated with 10 mM 2’-7’dichlorofluorescin diacetate (DCFH-DA) probes for 20 min before being exposed to rVvhA (500 ng/mL). As a well-established ROS-inducing agent, Rosup (50 μg/mL) was used as a positive control. At predetermined times (0, 0.5, 1, 2, 4, 6 h), the cells were harvested for fluorescence detection at excitation and emission wavelengths of 488 and 525 nm, respectively. To examine the protective effect of NSs, a toxin-containing culture medium was immediately incubated with NSs (40 μg/mL) for 30 min and then centrifugated at 10,000 rpm for 10 min. The supernatants were obtained and used to treat cells for 1 h, and then intracellular ROS levels were measured using flow cytometry or fluorescence microscopy.

For ATP detection, HPAECs were incubated with rVvhA (500 ng/mL) for various times (0, 1, 3, 6, 12 h), lysed and centrifuged at 14,000 rpm for 5 min at 4 °C. The supernatants were harvested, and the intracellular ATP concentration was measured using a luminometer and bioluminescence imaging. Next, toxins were pre-incubated with NSs in medium for 30 min, followed by centrifugation for harvesting supernatants. After that, HPAECs were treated with harvested supernatants for 12 h. Then, all the cells were harvested for fluorescence intensity detection via a luminometer or bioluminescence imaging. Cells treated with PBS or NSs served as controls.

### 4.5. Analysis of PKC/ERK/JNK and NF-κB Pathway in HPAECs

First, we explored the effects of rVvhA (500 ng/mL) on apoptosis-related signaling pathways. At predetermined time points (0, 15, 30, 60, and 90 min), rVvhA-treated HPAECs were harvested, washed, and then lysed to obtain cellular proteins to detect PKC-JNK-ERK and NF-κB pathways. Similarly, at different time points (0, 60, 90, 120, and 180 min), rVvhA-treated cells were harvested to detect apoptosis-related proteins. After determining the optimal exposure time, the effects of NSs (40 μg/mL) on apoptosis-related factors were then investigated. NSs were first pre-incubated with rVvhA (500 ng/mL) in RPMI 1640 medium for 30 min, followed by centrifugation to obtain supernatants. The cells were then treated with the supernatants and harvested at the optimal time point for apoptotic factor analysis.

Specifically, for Western blotting experiments, 20 micrograms of protein were resolved by 10% SDS-PAGE and transferred to a polyvinylidene difluoride membrane. The membrane was blocked with 5% skim milk for 2 h and incubated with the appropriate primary antibody (1:500) at 4 °C overnight. Next, the membrane was washed with TBST and incubated with a horseradish peroxidase-conjugated secondary antibody (1:2000) for 1 h. Subsequently, the protein bands were visualized by a ChemiDoc XRS+ System (Bio-Rad, Richmond, VA, USA) and quantified using ImageJ software (Version 1.8.0, NIH, Bethesda, MD, USA). For immunofluorescence staining, the cells were fixed for 10 min at room temperature, rinsed with PBS, blocked, and then incubated with NF-κB p65 antibodies overnight at 4 °C. After being washed with PBS three times, the cells were incubated with conjugated antibodies for 1 h at room temperature. After that, 4′,6-diamidino-2-phenylindole (DAPI) dye was added and incubated for 5 min before an anti-fluorescence quenching liquid was used and the cells were observed under a fluorescence microscope.

### 4.6. In Vivo rVvhA Detoxification by NSs

To analyze rVvhA-induced acute toxicity, male ICR mice (18–20 g) were randomly divided into five groups (*n* = 6), and different doses of rVvhA (3, 4, 5, 6, 7 mg/kg) were injected intravenously via the tail vein. The survival rate of each group was then recorded and analyzed. For systemic detoxification experiments, various amounts of NSs (0, 50, 100, 150, 200, 250 mg/kg) were injected via the tail immediately after exposure to a lethal intravenous dose of rVvhA (6 mg/kg), and the survival rate was recorded. For anti-inflammatory experiments, the mice were first injected with a sub-lethal dose of rVvhA (3.7 mg/kg, 0.75 × LD_50_). Then, at predetermined times (0, 3, 6, 12 h), the inflammatory factors, including IL-6 and TNF-α, were measured using ELISA kits. After that, the optimal time point (3 h), when rVvhA exerted the most obvious inflammatory injuries, was chosen to examine the inhibitory effects of NSs on toxin-induced inflammatory cascades. Finally, the mice were sacrificed, the lungs were harvested, and the tissues were fixed for H&E staining and pathological observations.

The local detoxification abilities of NSs against rVvhA were also tested. First, rVvhA (30 μg) was injected subcutaneously into the right flanks of mice to imitate local infections. Then, NSs (50 mg/kg) were injected immediately into the same regions to test their protective effects against rVvhA-induced wound injuries. An equal amount of PBS was used in the negative control group. After 3 d, the injection sites were measured and photographed, and then the topical skin and muscle tissues were harvested for H&E staining and pathological observations.

### 4.7. Statistical Analysis

The data are presented as the mean ± standard error (SE). Statistical significance was evaluated using one-way ANOVA (*p* < 0.05).

## 5. Conclusions

In conclusion, we synthesized a biocompatible nanoscale detoxification system (NSs) and used it for VvhA neutralization for the first time. NSs exerted significant protective effects on VvhA-induced damage both in vitro and in vivo, which may be associated with toxin sequestration and blockage of toxin-host cell interactions. These results provide new insights into how NS is a novel treatment strategy for the detoxification of PFTs from antibiotic-resisted *V. vulnificus*.

## Data Availability

Not applicable.

## Supplementary Materials

The following supporting information can be downloaded at: https://www.mdpi.com/article/10.3390/ijms23126821/s1.
