# Supplementary material for: Biomimetic Nanosponges Enable the Detoxification of Vibrio vulnificus Hemolysin
Source: Int J Mol Sci. 2022 Jun 19;23(12):6821. doi: 10.3390/ijms23126821 (PMC9224624; doi:10.3390/ijms23126821)
Supplement: Supplementary file 1 [file ijms-23-06821-s001.zip › ijms-1748750-supplementary.pdf]

Biomimetic Nanosponges Enable the Detoxification of *Vibrio vulnificus* Hemolysin

Shuaijun Zou, [1, †] Qianqian Wang, [1, †] Peipei Zhang,[2, ‡] Bo Wang, [1] Guoyan Liu, [1] Fuhai Zhang, [1] Jie Li, [1] Fan Wang, [1] Beilei Wang,[1,*] Liming Zhang[1,*]

[1]Department of Marine Biomedicine and Polar Medicine, Naval Special Medical Center, Naval Medical University, Shanghai 200433, China

[2]Department of Marine Biological Injury and Dermatology, Naval Special Medical Center, Naval Medical University, Shanghai 200052, China

[‡] These authors contributed equally to this work

[*] Corresponding Authors: lilly_wang@126.com (B.W.); lmzhang@smmu.edu.cn (L.Z.).

1. Materials and Methods

Expression and purification of recombinant VvhA (rVvhA)

The oligonucleotides were designed based on the genomic sequence of the *V. vulnificus* strain FJ03-X2 (GenBank KC821520) with the signal peptide sequence removed. The sequence was amplified by PCR (forward primer: 5’-CATATGCAAGAATATGTGCCGATTGTTGAG-3’ and reverse primer: 5’-TCTAGACTAGAGTTTGACTTGTTGTAATGT-3’) and then cloned into the His_6 tag expression vector pCZN1. After verification of the target sequence in the recombinant plasmids, the recombinant vectors were expressed in *Arctic-Express* (DE3) cells (Agilent Technologies). The bacteria were grown in LB-ampicillin medium at 37 °C until the cultures reached an OD_{600} between 0.6 and 0.8. Then, the bacteria were induced with 0.5 mM isopropyl-β-dithiogalactopyranoside (IPTG) for 4 h. Next, the cultures were harvested, lysed and examined by sodium dodecyl sulfate-polyacrylamide gel electrophoresis (SDS-PAGE). A Ni-IDA-Sepharose Cl-6B affinity chromatography column (Sigma-Aldrich) was used for protein purification, and the protein was verified by Western blotting. The residual endotoxin in the purified rVvhA was measured using ToxinSensor^{TM} Endotoxin Test Kit (GenScript, Nanjing, China). Finally, the activity of rVvhA was determined according to the hemolysis protocol in a 2% murine RBC suspension [1].

2. Results

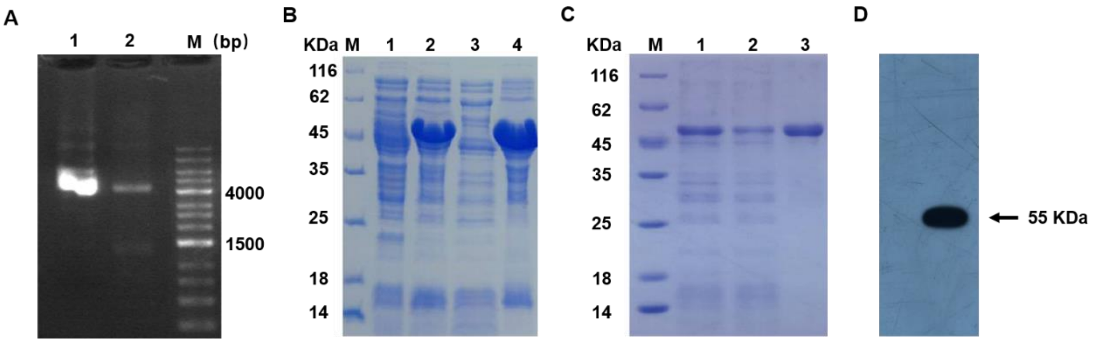

Figure S1. Expression and purification of rVvhA. (A) Determination of recombinant pCZN1 plasmids before and after enzyme digestion at *NdeI-XbaI* sites. (Line 1: plasmid before enzyme digestion, Line 2: plasmid after enzyme digestion, M: marker) **(B)** Determination of overexpression of rVvhA (M: marker, Line 1: sample from non-induced bacteria, Line 2: sample from IPTG-induced bacteria, Line 3: supernatant of lysed IPTG-induced bacteria, Line 4: sediment of lysed IPTG-induced bacteria). **(C)** SDS-PAGE analysis of rVvhA purification (M: marker, Line 1: sample harvested from lysed bacteria, Line 2: sample of outflow peak, Line 3: sample of elution peak). **(D)** Western-blot verification of purified rVvhA.

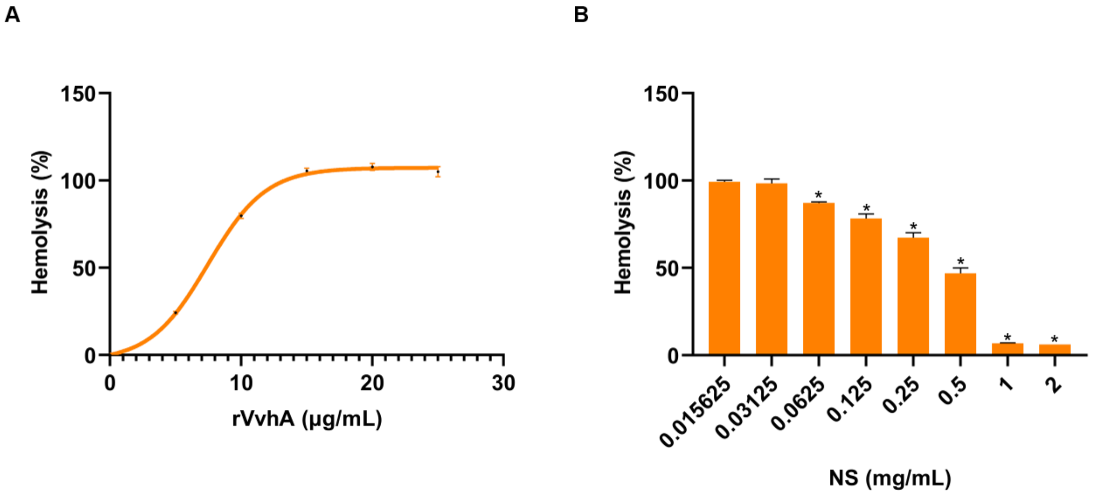

Figure S2. Hemolytic activity of rVvhA and inhibition effect of NSs. (A) Hemolytic activity of rVvhA on 2% RBC suspension *in vitro*. **(B)** Inhibition of NSs on rVvhA-caused hemolysis *in vitro*. (The data represent the means ± SE. n = 3. $^{*}p < 0.05$).

References

1. Zohra M.; Fawzia A. Hemolytic activity of different herbal extracts used in Algeria. *Int. J. Pharm. Sci. Res.* **2014**, *5*, 495-500.
